# Spatial distribution of tumor-infiltrating T cells indicated immune response status under chemoradiotherapy plus PD-1 blockade in esophageal cancer

**DOI:** 10.3389/fimmu.2023.1138054

**Published:** 2023-05-19

**Authors:** Cihui Yan, Hui Huang, Zhunhao Zheng, Xiaoxue Ma, Gang Zhao, Tian Zhang, Xi Chen, Fuliang Cao, Hui Wei, Jie Dong, Peng Tang, Hongjing Jiang, Meng Wang, Ping Wang, Qingsong Pang, Wencheng Zhang

**Affiliations:** ^1^ Department of Immunology, Tianjin Medical University Cancer Institute and Hospital, National Clinical Research Center for Cancer, Key Laboratory of Cancer Immunology and Biotherapy, Tianjin’s Clinical Research Center for Cancer, Tianjin, China; ^2^ Department of Radiation Oncology, Tianjin Medical University Cancer Institute and Hospital, National Clinical Research Center for Cancer, Key Laboratory of Cancer Prevention and Therapy, Tianjin’s Clinical Research Center for Cancer, Tianjin, China; ^3^ Department of Pathology, Tianjin Medical University Cancer Institute and Hospital, National Clinical Research Center for Cancer, Key Laboratory of Cancer Prevention and Therapy, Tianjin’s Clinical Research Center for Cancer, Tianjin, China; ^4^ Department of Endoscopy Diagnosis and Therapy, Tianjin Medical University Cancer Institute and Hospital, National Clinical Research Center for Cancer, Key Laboratory of Cancer Prevention and Therapy, Tianjin’s Clinical Research Center for Cancer, Tianjin, China; ^5^ Department of Nutrition Therapy, Tianjin Medical University Cancer Institute and Hospital, National Clinical Research Center for Cancer, Key Laboratory of Cancer Prevention and Therapy, Tianjin’s Clinical Research Center for Cancer, Tianjin, China; ^6^ Department of Esophageal Cancer, Tianjin Medical University Cancer Institute and Hospital, National Clinical Research Center for Cancer, Key Laboratory of Cancer Prevention and Therapy, Tianjin’s Clinical Research Center for Cancer, Tianjin, China; ^7^ Department of Lung Cancer, Tianjin Medical University Cancer Institute and Hospital, National Clinical Research Center for Cancer, Key Laboratory of Cancer Prevention and Therapy, Tianjin’s Clinical Research Center for Cancer, Tianjin, China

**Keywords:** esophageal squamous cell carcinoma, spatial analysis, immunotherapy, chemoradiotherapy, PD-1, tumor-infiltrating T cell

## Abstract

**Background:**

The spatial distribution of tumor-infiltrating T cells and its dynamics during chemoradiotherapy combined with PD-1 blockade is little known in esophageal squamous cell carcinoma (ESCC).

**Methods:**

We applied the multiplex immunofluorescence method to identify T cells (CD4^+^, CD8^+^ T cells, and their PD-1^−^ or PD-1^+^ subsets) and myeloid-derived cells (CD11c^+^ dendritic cells, CD68^+^ macrophages, and their PD-L1^+^ subpopulations) in paired tumor biopsies (*n* = 36) collected at baseline and during combination (40 Gy of radiation) from a phase Ib trial (NCT03671265) of ESCC patients treated with first-line chemoradiotherapy plus anti-PD-1 antibody camrelizumab. We used the FoundationOne CDx assay to evaluate tumor mutational burden (TMB) in baseline tumor biopsies (*n* = 14). We dynamically assessed the nearest distance and proximity of T-cell subsets to tumor cells under combination and estimated the association between T-cell spatial distribution and combination outcome, myeloid-derived subsets, TMB, and patient baseline characteristics.

**Findings:**

We found that the tumor compartment had lower T-cell subsets than the stromal compartment but maintained a comparable level under combination. Both before and under combination, PD-1^−^ T cells were located closer than PD-1^+^ T cells to tumor cells; T cells, dendritic cells, and macrophages showed the highest accumulation in the 5–10-μm distance. Higher CD4^+^ T cells in the tumor compartment and a shorter nearest distance of T-cell subsets at baseline predicted poor OS. Higher baseline CD4^+^ T cells, dendritic cells, and macrophages were associated with worse OS in less than 10-μm distance to tumor cells, but related with better OS in the farther distance. Higher on-treatment PD-1-positive-expressed CD4^+^ and CD8^+^ T cells within the 100-μm distance to tumor cells predicted longer OS. T cells, dendritic cells, and macrophages showed a positive spatial correlation. Both high TMB and smoking history were associated with a closer location of T cells to tumor cells at baseline.

**Conclusions:**

We firstly illustrated the T-cell spatial distribution in ESCC. Combining chemoradiotherapy with PD-1 blockade could improve the antitumor immune microenvironment, which benefits the treatment outcome. Further understanding the precision spatiality of tumor-infiltrating T cells would provide new evidence for the tumor immune microenvironment and for the combination treatment with immunotherapy.

## Introduction

Chemoradiotherapy (CRT) is the standard treatment strategy for patients with inoperable locally advanced esophageal squamous cell carcinoma (ESCC) (1, 2). However, survival remains poor for these patients. During the past 5 years, we carried out the first phase Ib clinical study of radiotherapy (RT) combined with the anti-PD-1 antibody camrelizumab in inoperable locally advanced ESCC (NCT03222440) (3) and then the first phase Ib clinical study of CRT plus camrelizumab as first-line treatment in these patients (NCT03671265) (4). Our ESCORT-CRT (NCT04426955) study and other studies, such as KEYNOTE-975 (NCT04210115) and RATIONALE 311 (NCT03957590), are phase III multicenter randomized clinical trials which are still ongoing combining CRT and anti-PD-1 antibody. The preliminary results showed that the 2-year overall survival (OS) rate was 69.6% in CRT plus PD-1 blockade (4). A fraction of the patients did not benefit from the combination treatment. Potential biomarkers are urgently needed in locally advanced ESCC patients treated with this combination treatment.

T cells, the major component of the adaptive immune response, play a vital role in immune surveillance and tumor control. T cells are important targets of checkpoint inhibitors and critical for the treatment outcome of immunotherapy in tumor therapy (5, 6). Recent studies revealed that T cells in treatment-naïve ESCC were always in a dysfunctional and exhausted status (7–9) which supported the deterioration of antitumor immune condition in locally advanced ESCC. We previously found that more tumor-infiltrating immune cells and less resident tumor cells during CRT predicted improved OS in locally advanced ESCC (10). In the phase Ib study of RT plus PD-1 blockade in locally advanced ESCC, we found that high PD-L1^+^CD4^+^ and PD-1^+^CD8^+^ T cells at baseline predicted poor OS (3). Meanwhile, we did not find an association between the total tumor-infiltrating T cells and survival in ESCC patients receiving CRT combined with PD-1 blockade (4). Multiregional sequencing illustrated the intratumor heterogeneity of the T-cell receptor (TCR) repertoire and its tight correlation with genomic mutation in ESCC (11). Single-cell sequencing also demonstrated the diverse signaling from heterogeneous stromal cells devoted to the divergent traits of immune cells in ESCC (7). Spatial analyses of immune cells in the tumor microenvironment would provide new evidence in immune-oncology research (12–14). However, precision spatial analysis of tumor-infiltrating T cells under CRT combined with immunotherapy is less studied so far.

Multiplex immunofluorescence uncovers the complicated interaction between multiple subpopulations in the tumor microenvironment (15–17). By using multiplex immunofluorescence, we previously identified the nearest distance of myeloid-derived cells, dendritic cells, and macrophages to tumor cells in the samples collected from the phase Ib clinical trial of CRT combined with camrelizumab (NCT03671265) (18). As a serial study, we here prospectively evaluated the nearest distance and proximity of T-cell subsets to tumor cells at baseline and on-treatment tumor tissues collected from these patients and its association with myeloid-derived cells, which provided precision biomarker candidates for predicting the outcome of CRT plus PD-1 blockade in ESCC.

## Materials and methods

### Study design

The phase Ib study evaluated the safety and feasibility of definitive CRT concurrently combined with anti-PD-1 antibody, camrelizumab, as the first-line treatment in inoperable locally advanced ESCC (NCT03671265) (4). Specifically, camrelizumab was given on day 1 of every 2-week period from the beginning of RT up to 32 weeks, concurrently with RT for 6 weeks, and with chemotherapy for 4 weeks (4). The exploratory endpoints of this phase Ib study were local and systematic immune characteristics and potential predictive biomarkers for treatment outcome.

### Sample collection

Tumor biopsies were collected before (baseline, within 3 days before treatment, *n* = 20) and under combination (on-treatment, after 40 Gy of radiation and two rounds of camrelizumab treatment, *n* = 18) (**Additional File: Table S1**). Deep biopsy samples of tumor tissues were collected under endoscopic ultrasonographic guidance (19, 20) and made into formalin-fixed paraffin-embedded (FFPE) tissue blocks.

### Ethics statement

This study conformed to the ethical principles outlined in the Declaration of Helsinki, and the protocol was approved by the Institutional Review Board and Ethics Committee of Tianjin Medical University Cancer Institute & Hospital (E2018142). All patients provided written informed consent to participate. This study was registered on ClinicalTrials.gov (NCT03671265).

### Multiplex immunofluorescence staining

To comprehensively describe the tumor immune microenvironment, we used the serial FFPE slides of the biopsy specimens collected at baseline or during the combination in multiplex immunofluorescence staining. The FFPE slides were 4 μm thick so monolayer cells could be identified in the following imaging analysis. The multiplex immunofluorescence staining was automatically performed in a Bond III automated stainer (Leica, USA). The TSA 5-color kit (#D110051-50T) and TSA 670 (#D110016-100T) were bought from Yuanxibio, Shanghai, China. Two staining panels were applied. The staining order was as follows: panel 1—anti-CD4 (#YX32005, Yuanxibio, 1:300)/TSA 620, anti-CD8 (#YX63005, Yuanxibio, 1:300)/TSA 670, anti-panCK (#GM351507, GeneTech, Shanghai, China, 1:6)/TSA 520, and anti-PD-1 (#10377-MM23, Sino Biological, Beijing, China, 1:200)/TSA 570; panel 2—anti-PD-L1 (#13684, CST, Danvers, USA, 1:800)/TSA 570, anti-panCK (#GM351507, GeneTech, 1:6)/TSA 520, anti-CD11c (#45581, CST, 1:300)/TSA 620, and anti-CD68 (#GM087602, GeneTech, ready-to-use)/TSA 670. To visualize the cell nuclei, the tissue was stained with 4′,6-diamidino-2-phenylindole (D1306; Thermo Fisher, Waltham, USA).

### Imaging and analysis

We performed a whole slide scan for each fluorescence-stained slide by using the digital microscopy scanner Pannoramic MIDI tissue imaging system (3DHISTECH Ltd., Hungary). Because the surface marker CK was expressed on both tumor and normal epithelial cells, it is hard to distinguish these two types of cells in immunofluorescence staining. We applied hematoxylin and eosin (H&E) staining in the tissue sections after finishing the panel 2 fluorescence scan in order to exclude the normal epithelial cells in the analysis. At the same time, we could clearly identify the tumor and normal epithelial cells on the serial tissue section for panel 1 staining according to the tumor cells judged by H&E staining in the serial slide. Images were analyzed by the HALO software (Version 3.5.3577, Indica Labs, Albuquerque, NM, USA). An experienced pathology specialist supervised to segment the tumor and stromal compartment using the HALO software. HALO Highplex FL (version 4.1) was used for nuclear segmentation and tumor-infiltrating lymphocyte quantification. In brief, all nuclei in the whole slide image were automatically segmented based on DAPI staining. Positivity thresholds for each marker were set based on cytoplasmic or nuclear staining intensity and were reviewed across all samples. Data for each cell’s expression of all the markers and the *x* and *y* locations within the tissue were stored in HALO for spatial analysis. Finally, cells were phenotyped as follows: panel 1—CD4^+^ T cell, CD8^+^ T cell, CD4^+^PD-1^+^ T cell, CD4^+^PD-1^−^ T cell, CD8^+^PD-1^+^ T cell, CD8^+^PD-1^−^ T cell, and tumor cell (panCK^+^); panel 2—dendritic cell (CD11c^+^), macrophage (CD68^+^), tumor cell (panCK^+^), and PD-L1^+^ subpopulations of these cells.

Immune cell infiltration was evaluated as the number of cells per whole slide, in the tumor compartment, stromal compartment, or individual regions of the slides, respectively. Spatial analysis was performed using the HALO Spatial Analysis module. To better evaluate the position relationship between immune cells and tumor cells, two algorithms, namely, the HALO nearest neighbor analysis and proximity analysis, were used. In analyzing the nearest distance, the distance between each tumor cell (as the core cell) and its nearest neighbor immune cells (CD4^+^, CD8^+^, CD4^+^PD-1^+^, CD4^+^PD-1^−^, CD8^+^PD-1^+^, and CD8^+^PD-1^−^ T cells) was measured. In the proximity analysis, total immune cells detected in each 5-μm interval within the 100-μm distance to each tumor cell (as the core cell) were counted.

### Tumor mutational burden test

To investigate the tumor mutation at baseline, the biopsy specimens from 14 patients before the combination treatment were sequenced by using FoundationOne CDx (F1CDx) and the FDA-approved 324-gene panel assay conducted by DIAN (Hangzhou Lab, Hangzhou, China) with licensed technologies, to assess the tumor mutational burden (TMB) (**Additional File: Table S1**) (21).

### Statistical analyses

Non-parametric two-sided Mann–Whitney *U* test or the Wilcoxon signed-rank test was used to evaluate the statistical significance between two independent or paired groups, respectively. The Kruskal–Wallis test was used to estimate the statistical significance for comparing more than two groups. Non-Gaussian distribution (Spearman correlation) was applied to assess the correlations unless otherwise indicated. OS was defined as the time from inclusion until death from any cause or the last date of follow-up time. Progression-free survival (PFS) was defined as the time from inclusion until the date of objective disease progression or death from any cause in the absence of progression. The Kaplan–Meier analysis was used to estimate OS and PFS. Differences in survival were compared with the log-rank tests. The best cutoff of the Kaplan–Meier survival analysis was calculated by the Youden index of the ROC curve.

Analyses were performed using SPSS v.25.0 (STATA, College Station, TX, USA). All statistical tests were two-sided, and the significance level was set at 0.05. Survival curves and summary graphs were presented using GraphPad Prism v.8.0. The relevance between T cells, dendritic cells, and macrophages was developed using R version 4.2.2. The data cutoff date for all analyses was 30 August 2022.

## Results

### Different T-cell infiltration between the tumor and stromal compartment

**Figure 1:**
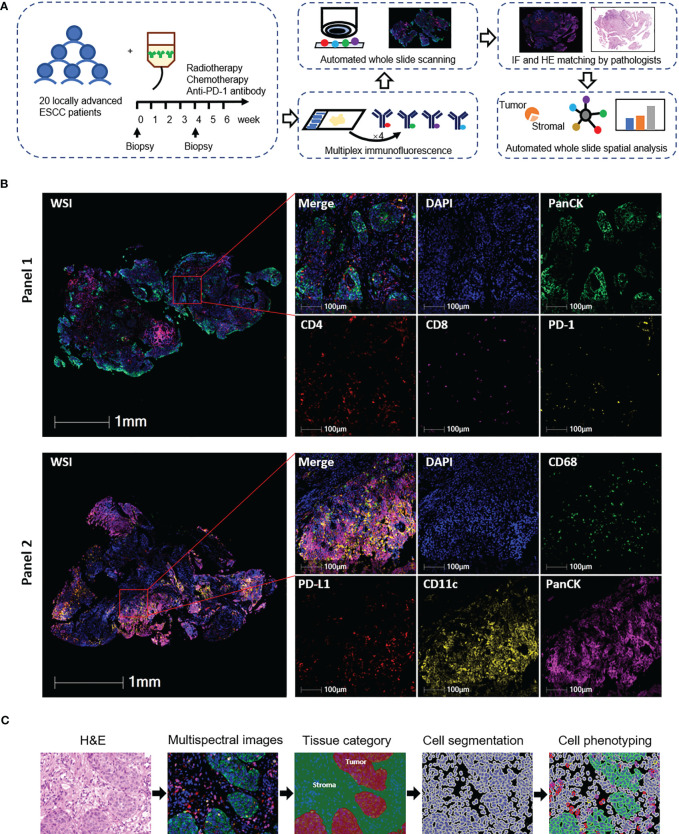
Identification of tumor-infiltrating T cells, dendritic cells, and macrophages in esophageal squamous cell carcinoma (ESCC) tissues. **(A)** Schematic diagram of the study design and analysis process. Top middle, representative panel 1 staining of baseline tumor tissue from patient 18. Top right, representative panel 2 staining of baseline tumor tissue from patient 8. **(B)** Representative images of multiplex immunofluorescence panels. WSI, whole slide imaging. From patients 6 (panel 1) and 19 (panel 2). **(C)** Automated image analysis pipeline. From patient 18 baseline tumor tissue.

Twenty treatment-naive patients were finally included in the phase Ib study (NCT03671265, **Additional File: Table S2**). To explore the spatial characteristics of T cells, dendritic cells, and macrophages and their dynamic alteration in ESCC patients under the combination treatment of CRT plus PD-1 blockade, we applied multiplex immunofluorescence staining in 38 tumor biopsies collected at baseline or during the combination treatment (**Figures 1A, B**; **Additional File: Table S1**). We firstly excluded two baseline slides without tumor tissues and finally included 36 scanned slides in the following analysis, consisting of 18 baseline and 18 on-treatment specimens, with 16 matched pairs at these two time points (**Additional File: Table S1**).

With the updated data cutoff date of 30 August 2022, the median follow-up duration was 44.3 months (95% CI 41.8–46.7). Seven patients were alive and free of progressive disease. The median OS and PFS were 36.8 months (95% CI 22.2 to 42.3) and 31.9 months (95% CI 15.9–42.3), respectively (**Additional File: Figure S1**).

**Figure 2:**
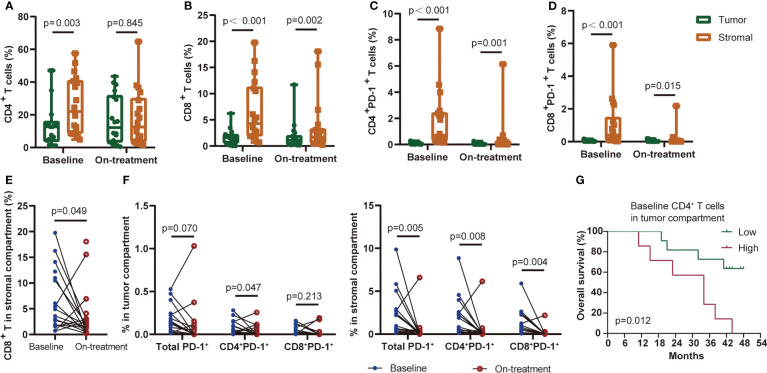
T-cell subsets distributed in the tumor or stromal compartment. CD4^+^ T cells **(A)**, CD8^+^ T cells **(B)**, CD4^+^PD-1^+^ T cells **(C)**, and CD8^+^PD-1^+^ T cells **(D)** distributed in the tumor or stromal compartment at baseline and during treatment. **(E)** Baseline and on-treatment CD8^+^ T cells in the stromal compartment. **(F)** PD-1-positive-expressed T cells in the tumor (left) or stromal (right) compartment at baseline and during the combination treatment. **(G)** Kaplan–Meier curve showing overall survival based on CD4^+^ T cells in the tumor compartment at baseline. Cutoff value: 13.97%. On-treatment, after 40 Gy of radiotherapy. Wilcoxon signed-rank tests in **(A-F)**. *p* < 0.05, statistically significant.

We previously reported the comparable levels of CD4^+^ and CD8^+^ T cells before and during CRT combined with PD-1 blockade in ESCC patients (4). Since T cells located in the tumor and stromal area had various features and capabilities in immune response (22, 23), we here segmented the tumor tissues into tumor and stromal compartments to identify T-cell levels in these two compartments (defined as tumor T cells and stromal T cells, respectively) (**Figure 1C**). The level of tumor CD4^+^ T cells was lower than that of stromal CD4^+^ T cells at baseline, while it was not different during combination (**Figure 2A**). The levels of tumor CD8^+^ T cells, tumor PD-1^+^CD4^+^, and tumor PD-1^+^CD8^+^ T cells remained lower than those in stromal cells both at baseline and during combination treatment (**Figures 2B–D**). Next, we dynamically monitored the T-cell levels in these two compartments. CRT plus PD-1 blockade did not affect the levels of tumor CD4^+^ and tumor CD8^+^ T cells. However, the stromal CD8^+^ T cells and stromal PD-1^+^ T cells decreased significantly after combination treatment (**Figures 2E, F**). Finally, we assessed the association between each T-cell subset and patient survival. The patients having higher tumor CD4^+^ T cells at baseline had worse OS and worse PFS compared with those having lower baseline tumor CD4^+^ T cells (**Figure 2G**; **Additional File: Figure S2**). These results indicated the inhibitory immune microenvironment in ESCC tumors before treatment, and CRT combined with PD-1 blockade alleviated the repressive immune status which might benefit the treatment outcome.

### Nearest distance of T cells to tumor cells

**Figure 3:**
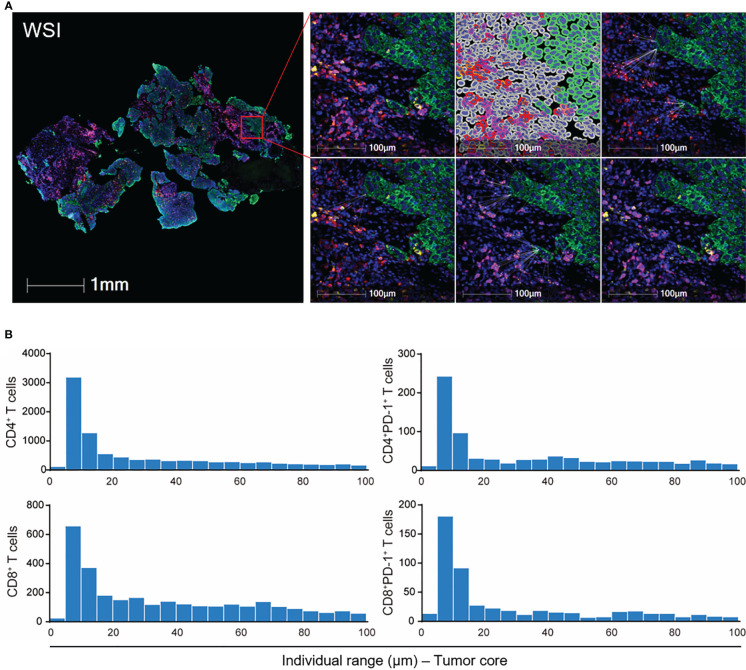
Spatial analysis of tumor-infiltrating T cells in ESCC. **(A)** Schematic graph of the nearest distance analysis. Green: tumor cells. Red: CD4^+^ T cells. Purple: CD8^+^ T cells. Yellow: PD-1^+^ cells. Upper right panel: from left to right, merged fluorescence, cell phenotyping, nearest neighbors of CD4^+^ T cells to tumor cells. Lower right panel: from left to right, nearest neighbors of CD4^+^PD-1^+^, CD8^+^, and CD8^+^PD-1^+^ T cells to tumor cells. **(B)** Proximity analysis in WSI in **(A)**. WSI, whole slide imaging. From patient 18 baseline tumor tissue.

Since tumor-infiltrating T cells exhibited different compartmental distribution (tumor *vs.* stromal compartment) (**Figure 2**), we then focused on the spatial distribution of tumor-infiltrating T cells as it more precisely reflected the regional tumor immune microenvironment and the probability of interaction between the effective T cells and target tumor cells (**Figure 3**). We applied two types of spatial parameters. The nearest distance demonstrated possible interaction between the nearest neighbors of the T cells and tumor cells by integrating the spatial distribution and count number traits of T cells in the tumor microenvironment (**Figure 3A**). The proximity between T cells and tumor cells illustrated not only the heterogeneous spatial location of T cells but also the heterogeneous tumor immune microenvironment (**Figure 3B**).

**Figure 4:**
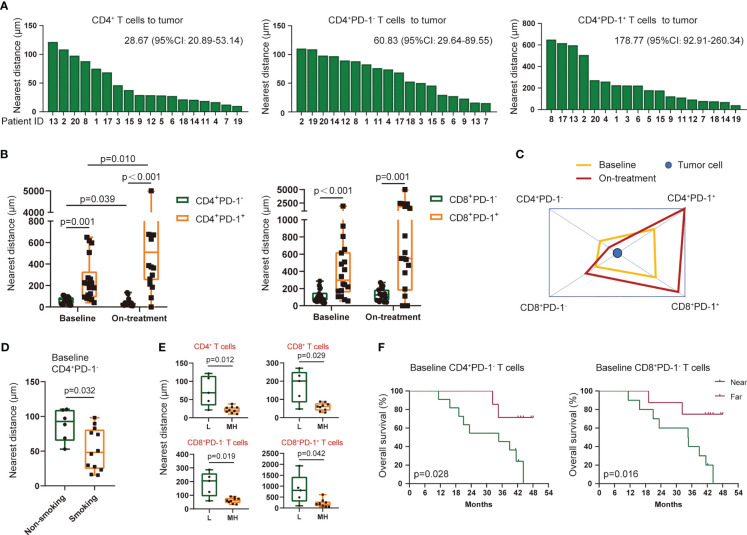
Nearest distance of T-cell subsets to tumor cells. **(A)** Nearest distance of CD4^+^ T cells (left), CD4^+^PD-1^−^ T cells (middle), and CD4^+^PD-1^+^ T cells (right) to tumor cells for each patient. Statistics: median (95% confidence interval). Arranged by distance from high to low. **(B)** Comparation of the nearest distance to tumor cells between CD4^+^PD-1^−^ and CD4^+^PD-1^+^ T cells and between CD8^+^PD-1^−^ and CD8^+^PD-1^+^ T cells at baseline and under the combination treatment. **(C)** Schematic diagram of the nearest distance of T-cell subsets to tumor cells. CD4^+^PD-1^+^ and CD8^+^PD-1^+^ T cells had the farthest distance from tumors both at the two time points. **(D)** Nearest distance of baseline CD4^+^PD-1^−^ T cells to tumor cells between patients with and without a smoking history. **(E)** Nearest distance of T-cell subsets to tumor cells between patients with low and middle/high TMB at baseline. **(F)** Kaplan–Meier curve showing the overall survival based on the nearest distance of baseline CD4^+^PD-1^−^ T cells and baseline CD8^+^PD-1^−^ T cells to tumor cells. Cutoff value: 79.58 μm for CD4^+^PD-1^−^ T cells and 95.88 μm for CD8^+^PD-1^−^ T cells. Wilcoxon signed-rank tests in **(B)**. Kruskal–Wallis tests used among subsets at baseline and under the combination treatment in **(C)**, respectively. Mann–Whitney *U* tests in **(D, E)**. L, TMB low (≤5 Muts/Mb); MH, TMB middle or high (>5 Muts/Mb); on-treatment, after 40 Gy of radiotherapy. *p* < 0.05, statistically significant.

T-cell subsets had diverse nearest distances to tumor cells among each patient (**Figure 4A**, **Additional File: Figure S3**). The PD-1-negative-expressed CD4^+^ and CD8^+^ T cells were located significantly nearer to the tumor cells than PD-1-positive-expressed T cells (**Figure 4B**). Moreover, CD4^+^PD-1^−^ T cells migrated closer to the tumor cells, while CD4^+^PD-1^+^ T cells moved even farther away from the tumor cells under the combination treatment (**Figures 4B, C**). A higher density of tumor-infiltrating CD8^+^ T cells was reported in heavy smokers with advanced non-small cell lung cancer (24). We also found that ESCC patients with a smoking history had a shorter distance of CD4^+^PD-1^−^ T cells to tumor cells before treatment than those without (**Figure 4D**). However, the other baseline characteristics of the patients, such as aging, gender, and alcohol, were not associated with the nearest distance of T-cell subsets to tumor cells. Interestingly, we found a close association between higher TMB and smaller nearest distance of CD4^+^ and CD8^+^ T cells to tumor cells in baseline tumor tissues (**Figure 4E**). This association was in accordance with the PD-1-negative- or PD-1-positive-expressed CD8^+^ T cells (**Figure 4E**), and this turned marginal in CD4^+^PD-1^+^ T cells before combination treatment (*p* = 0.060, **Additional File: Figure S4**). In the survival analysis, we found that patients who had CD4^+^ and CD8^+^ T cells or their PD-1^−^ subsets located nearer to tumor cells at baseline experienced worse OS and PFS (**Figure 4F**, **Additional File: Figure S5**). These findings again suggested that these tumor-infiltrating T cells were inhibited in ESCC before CRT combined with PD-1 blockade, and spatialized T cells with distinctive PD-1 expression were linked with the heterogenicity of the tumor microenvironment.

### Proximity of T cells in ESCC

**Figure 5:**
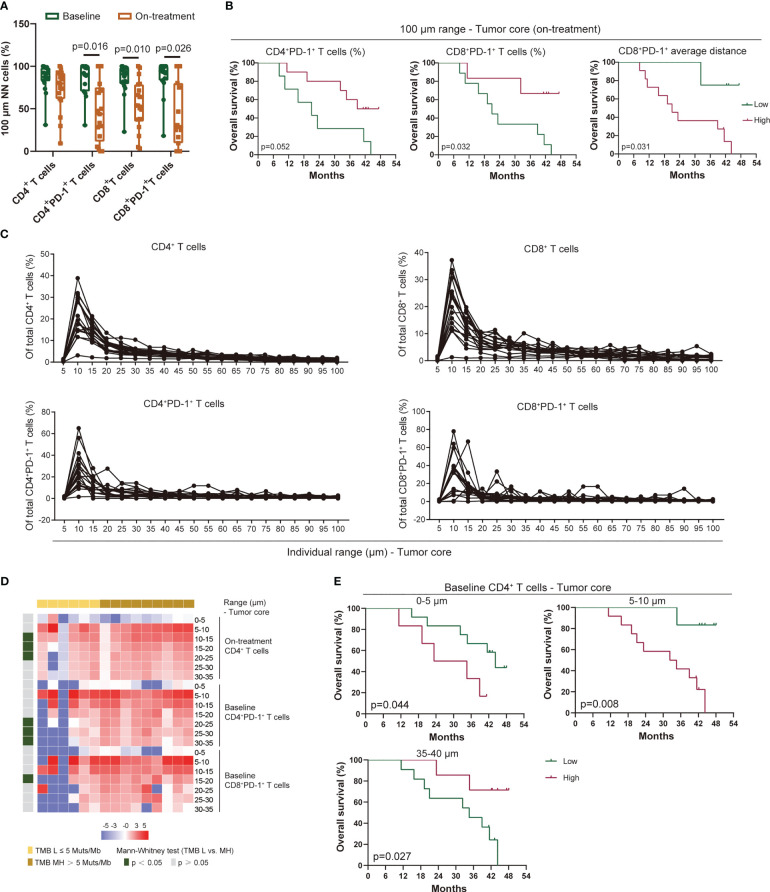
Proximity of T-cell subsets in ESCC. **(A)** T-cell subsets within the 100-μm distance to tumor cells. **(B)** Kaplan–Meier curve showing the overall survival based on the proportion of CD4^+^PD-1^+^ and CD8^+^PD-1^+^ T cells and the average distance of CD8^+^PD-1^+^ T cells within the 100-μm distance to tumor cells during treatment. Cutoff value: 31.51% for CD4^+^PD-1^+^ T cells and 38.72% for CD8^+^PD-1^+^ T cells; 47.75 μm for the average distance of CD8^+^PD-1^+^ T cells. **(C)** Proportion of T-cell subsets in each 5-μm range across the 100-μm distance to tumor cells at baseline. **(D)** Heatmap illustrating the association between TMB and the proportion of T-cell subsets in individual distance to tumor cells. **(E)** Kaplan–Meier curve showing the overall survival based on the proportion of CD4^+^ T cells in the 0–5-, 5–10-, and 35–40-μm distance to tumor cells at baseline. Cutoff values: 0.97%, 14.95%, and 3.81%. On-treatment, after 40 Gy of radiotherapy. Wilcoxon signed-rank tests in **(A)**. Mann–Whitney *U* test in **(D)**. *p* < 0.05, statistically significant.

To further evaluate the position of T cells in each tumor cell, we narrowed the observation area within the 100-μm radius of the tumor cells and assessed T cells in a continuous 5-μm distance away from the tumor cells (**Figure 3B**). The baseline CD4^+^, CD8^+^ T cells, and their PD-1-positive-expressed subsets were mostly located within the 100-μm distance to tumor cells (**Figure 5A**). Under the combination treatment, part of these cells dispersed out of the 100-μm distance (**Figure 5A**). Higher PD-1-positive-expressed CD4^+^ and CD8^+^ T cells within the 100-μm distance during treatment predicted better OS (**Figure 5B**, **Additional File: Figure S6**). The average distance within the 100-μm distance of T-cell subsets to tumor cells also indicated an underlying interaction between T cells and tumor cells. In our study, a smaller average distance of the on-treatment CD8^+^PD-1^+^ T cells within the 100-μm distance to the tumor cells was associated with longer OS and PFS (**Figure 5B**, **Additional File: Figure S6**). These findings exhibited an improved antitumor immune microenvironment under CRT plus PD-1 blockade.

In analyzing the distribution pattern of T-cell subsets in each 5-μm range across the 100-μm distance to tumor cells, we defined the relative density of T-cell subset A as the number of T-cell subset A in one range divided by the total T-cell subset A detected in tumor tissue. We found few CD4^+^ and CD8^+^ T cells infiltrated in the 0–5-μm distance both at baseline and during combination treatment (**Figure 5C**, **Additional File: Figure S7**). Meanwhile, these T cells increased sharply in the 5–10-μm range, then decreased gradually in the 10–30-μm range, and finally maintained at a low level beyond the 30-μm distance (**Figure 5C**, **Additional File: Figure S7A**). A similar distribution pattern in PD-1-positive-expressed CD4^+^ and CD8^+^ T cells was also observed at baseline and during treatment (**Figure 5C**, **Additional File: Figure S7B**). Because the imbalanced expression of PD-1 on T cells might influence the distribution pattern of PD-1^+^ T cells, we assessed the ratio of PD-1^+^ T cells to T cells in each 5-μm range across the 100-μm distance. However, both CD4^+^ and CD8^+^ T cells remained at a stable level of PD-1 expression among each range at baseline although heterogeneity was also found among individual patient (**Additional File: Figure S8A**). The on-treatment PD-1 expression on T cells remained at a low level across each range in the 100-μm distance except for one patient who had a large fluctuation of PD-1 expression (**Additional File: Figure S8B**). We did not find any association between the distribution traits of these T-cell subsets in the 100-μm distance and the patient baseline characteristics (aging, gender, alcohol, and smoking). However, patients with higher TMB had higher baseline CD4^+^PD-1^+^ T cells, baseline CD8^+^PD-1^+^ T cells, and on-treatment CD4^+^ T cells in the 10–35-μm distance (**Figure 5D**). In the survival analysis, we found that baseline CD4^+^ T cells in the 0–5- and 5–10-μm distance were correlated with poor OS and PFS, but this association was reversed in the 35–40-μm range (**Figure 5E**, **Additional File: Figure S9**). These findings illustrated a precise spatial distribution of tumor-infiltrating T cells in ESCC, which presented a heterogeneous interaction between regional T cells and tumor cells.

### Proximity of DCs and macrophages in ESCC

**Figure 6:**
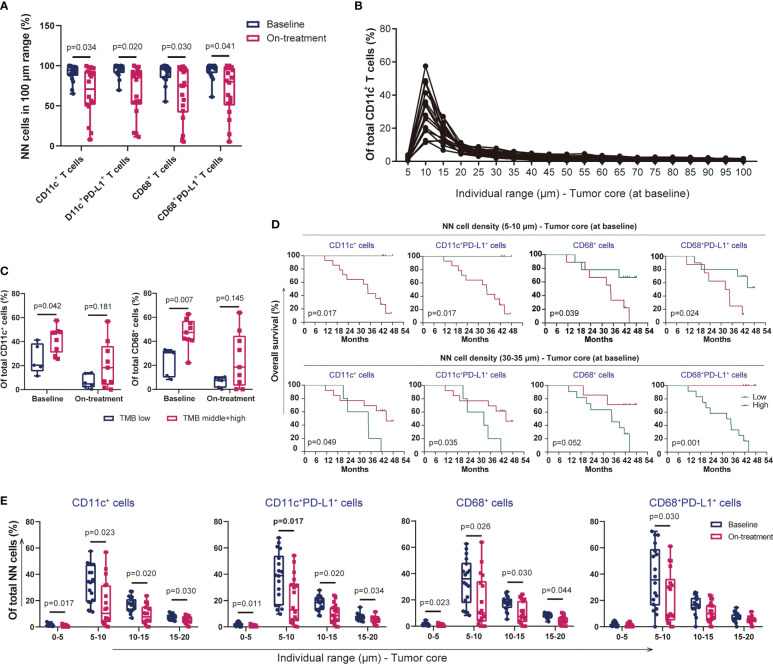
Proximity of dendritic cells and macrophages in ESCC. **(A)** CD11c^+^ dendritic cells, CD68^+^ macrophages, and their PD-L1^+^ subsets within the 100-μm distance to tumor cells. **(B)** Proportion of CD11c^+^ dendritic cells in each 5-μm range across the 100-μm distance to tumor cells at baseline. **(C)** Dendritic cells and CD68^+^ macrophages within the 100-μm distance to tumor cells in patients with low or middle/high TMB. **(D)** Kaplan–Meier curve showing the overall survival based on the proportion of myeloid-derived cells in the 5–10- and 30–35-μm distance to tumor cells at baseline. Cutoff values: 18.79%, 16.14%, 20.91%, and 40.89% in the 5–10-μm distance; 2.75%, 2.11%, 3.55%, and 3.40% in the 30–35-μm distance. Similar groups divided according to the cutoff values of 18.79% and 16.14% in CD11c^+^ and CD11c^+^PD-L1^+^ cells in the 5–10-μm distance. Consequently, the survival curves and significant differences were the same (two graphs in the top left corner). **(E)** Decreased proportion of myeloid-derived cells within the 20-μm distance to tumor cells under the combination treatment. On-treatment, after 40 Gy of radiotherapy. Wilcoxon signed-rank tests in **(A)** and **(E)**. Mann–Whitney *U* test in **(C)**. *p* < 0.05, statistically significant.

We previously reported the nearest distance of myeloid-derived immune cells, CD11c^+^ DCs, and CD68^+^ macrophages and its close association with outcome under CRT plus PD-1 blockade (18). We here evaluated the precision distribution of these cells in ESCC. Similar to the findings in T cells (**Figure 5**), the majority of DCs, macrophages, and their PD-L1+ subsets distributed at the 100-mm distance to the tumor cells (**Figure 6A**). A high proportion of these subsets was located between the 5- and 30-μm distance, with the highest in the 5–10-μm range at baseline and during combination (**Figure 6B**, **Additional File: Figure S10**). Both higher DCs and higher macrophages in the 5–10-μm distance at baseline were associated with higher TMB, and the correlation tended to have significance during combination (**Figure 6C**). DCs and macrophages exhibited an evenness level of PD-L1 expression across the 0–100-μm distance in each patient (**Additional File: Figure S11**). The myeloid-derived cells distributed at different distances to tumor cells had divergent associations with patient survival. Patients with lower subsets of DCs and macrophages at a nearer distance (0–5 and 5–10 μm) at baseline had shorter OS and PFS (**Figure 6D**, **Additional File: Figure S12**). On the contrary, patients with higher subsets of these cells in relatively farther distances (20–25, 25–30, and 30–35 μm) at baseline had longer OS and PFS (**Figure 6D**, **Additional File: Figure S13**). We found one exception between T and myeloid-derived cells. The myeloid-derived cells had declined distribution in the 5–10-, 10–15-, and 15–20-μm range during combination compared with those at baseline (**Figure 6E**), but there was no alteration in T cells in any range across the 100-μm distance during combination. The similar distribution patterns of T cells, DCs, and macrophages to tumor cells strongly indicated the potential association between these cells in ESCC.

### Association between T cells, DCs, and macrophages in ESCC

**Figure 7:**
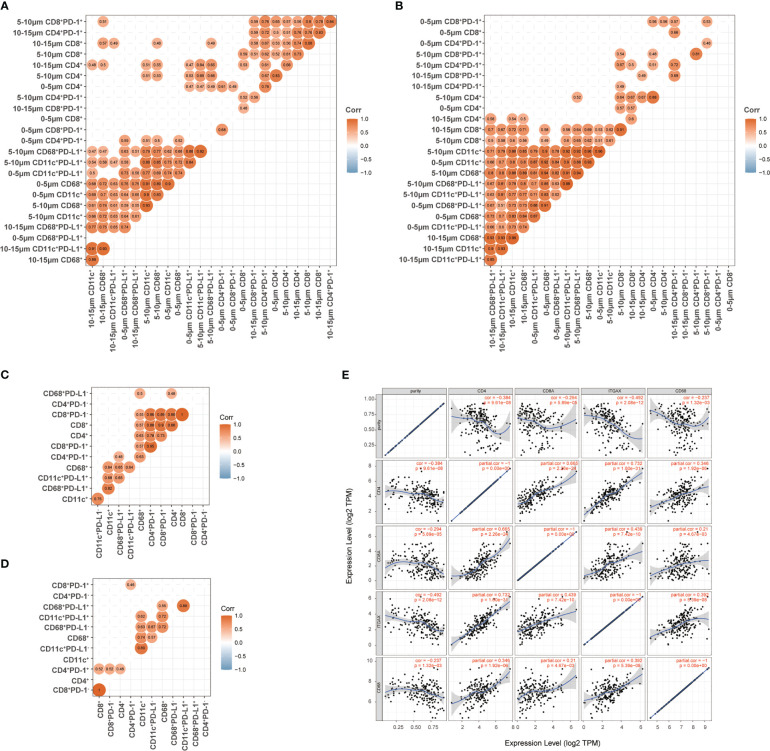
Association between T cells, dendritic cells, and macrophages in ESCC. **(A, B)** Association between the proportion of T cells and the proportion of dendritic cells and macrophages in the 0–15-μm distance to tumor cells at baseline **(A)** and during treatment **(B)**. **(C, D)** Association between the nearest distance of T cells and the nearest distance of dendritic cells and macrophages to tumor cells at baseline **(C)** and during treatment **(D)**. **(E)** TIMER2.0 evaluated the correlation between T cells and myeloid-derived cells in esophageal cancer. Spearman correlation in **(A-D)**. Correlation coefficient was shown when *p <*0.05 with statistical significance.

We firstly determined the relationship between the subsets of T cells, DCs, and macrophages in the 0–15-μm distance to the tumor cells, as a large part of these cells were located in this area and had the most possibility to interact with tumor cells directly. Before the combination treatment, the strongest correlation was observed between DCs and macrophages and their PD-L1-positive subsets, moderate correlation between T-cell subsets, and low correlation between the T and DC/macrophage subsets (**Figure 7A**). After the combination treatment, the correlation between the DC/macrophage subsets was even higher than that at baseline, and the correlation between the CD8^+^ T and DC/macrophage subsets also improved (**Figure 7B**). Meanwhile, we evaluated the relevance of the nearest distance between the T-cell and DC/macrophage subsets. High relevance was found between T-cell subsets and moderate between DC/macrophage subsets at baseline (**Figure 7C**). Macrophages were more relevant than DCs with the T-cell subsets at baseline (**Figure 7C**). After the combination treatment, the T-cell subsets became weakly correlated, while DCs and macrophages still had a strong correlation (**Figure 7D**). Finally, we used TIMER2.0 (http://timer.cistrome.org/) (25) to evaluate the correlation between T cells, DCs, and macrophages in esophageal cancer and found a consistent correlation between these cells (**Figure 7E**).

## Discussion

In the present study, we precisely depicted the inhibitory status of T cells in locally advanced ESCC by using spatial analysis. We found an improved antitumor immune microenvironment under CRT plus PD-1 blockade, which predicted better treatment outcome. Our results also revealed the significant spatial connection between T cells and myeloid-derived cells, which was consistently maintained throughout the combination treatment. TMB and smoking exposure promoted T cells to accumulate near tumor cells in ESCC.

We found that tumor CD4^+^ T cells and the nearest distance of CD4^+^PD-1^−^ and CD8^+^PD-1^−^ to tumor cells at baseline predicted poor survival in ESCC patients. Recent evidence revealed an inhibitory and exhausted status of T-cell subsets in the immune microenvironment of ESCC (7–9). The heterotypic interaction between cancer cells and non-cancer cells of the tumor microenvironment remolded a tumor-supportive and immune-repressive environment. Direct intercellular contact and paracrine signaling both contributed to the dysfunctional status of immune cells. The inhibitory induction factors might be derived from tumor cells (26, 27), abnormally regulated immune and stromal cells in the tumor microenvironment (28, 29), and distant endocrine cytokines (30), as well as metabolic changes in tumor cells and non-cancer cells. We found that the tumor compartment had lower T cells than the stromal compartment, and PD-1^+^ T cells were located farther away from the tumor cells compared with the PD-1^−^ T cells at baseline. The PD-1^+^ T cells might be composed of more tumor-specific T cells in the tumor microenvironment (31, 32). These results indicated that the potential tumor-specific T cells were probably secluded from the tumor cells at a distance in locally advanced ESCC without any therapeutic intervention. This spatial distribution pattern of T-cell subsets in ESCC accelerated the loss of immune surveillance and promoted immune escape. We found that high levels of CD4^+^ T cells distributed in the 0–10-μm distance to tumor cells at baseline predicted poor survival. ESCC has been shown to harbor tumor-infiltrating Foxp3^+^CD4^+^ regulatory T cells (Tregs) (33, 34). However, the infiltrating Tregs could not predict survival in ESCC patients (35). Various bystander T cells that recognized a wide range of epitopes unrelated to cancer accumulated in tumor tissues but had no antitumor efficiency (36). Identifying subgroups and tumor specificity of infiltrating T cells could provide new evidence for the underlying mechanisms of immune response. All these findings supported a deteriorated antitumor immune microenvironment in locally advanced ESCC, which was partially characterized by the dysfunction and distance barrier for the tumor-infiltrating T cells with potential antitumor capability.

We found comparable levels of T-cell subsets in the tumor compartment before and after the combination treatment. T cells were more sensitive to CRT compared with myeloid-derived cells (37). Hematological toxicity was always one of the most serious side effects of CRT or CRT plus PD-1 blockade (1, 4). However, we indeed found clonal expansion of tumor-infiltrating T cells that had already existed in local ESCC tumors before the combination treatment of CRT plus PD-1 blockade (38). Meanwhile, more than 90% of T-cell clones in on-treatment tumors were newly acquired after the combination treatment (38). The peripheral blood provided an abundant T-cell pool to support the local antitumor immune response (39, 40). A recent study also revealed that tumor-specific memory CD8^+^ T cells in draining lymph nodes respond to PD-1/PD-L1 blockade (5). CRT disturbed the suppressive tumor immune microenvironment and improved the antitumor immune response, which was further strengthened with the addition of PD-1 blockade. Taking these results together with our findings into consideration, it can be inferred that combining PD-1 blockade rejuvenated and recruited T cells to the tumor microenvironment, which enriched the tumor-infiltrating T cells and contributed to improved treatment outcome.

We found that high PD-1-positive-expressed CD4^+^ and CD8^+^ T cells within the 100-μm distance to the tumor cells during the combination treatment were significantly associated with better OS. The tumor-infiltrating PD-1^+^ T cells during treatment indicated activated status and antitumor potential, although the specificity of these cells was unavailable in the present study. In our findings, most of the T cells, DCs, and macrophages were distributed within the distance of 5–30 μm, especially in the 5–10-μm distance. However, our results did not show that the high density of T/DCs/macrophages within the 10-μm distance had an effect on tumor control. In contrast, those distributed in the 20-μm or farther distance were probably the antitumor candidates. Interleukin-33 induced CCL2 and IL-7 induced CXCL9, CXCL10, CCL2, and CCL20 expression in ESCC tissues (41, 42). CD4^+^, CD8^+^ T cells, macrophages, and DCs expressing the receptors of these chemokines were recruited into ESCC (41–44), where these cells were regulated by complex signaling from the regional tumor microenvironment and presented diverse capabilities participating in pro- or antitumor immune response. These results enlightened the spatial and functional heterogenicity of tumor-infiltrating immune cells critically and were diversely devoted to the treatment outcome of CRT plus PD-1 blockade. We are now working on the precise mechanisms that inhibit or activate immune cells in these heterogeneous tumor regions before and under the combination treatment.

CRT promoted the release of danger signals and chemokines that recruited DCs and macrophages into tumor sites and provoked tumor cell killing by activating cytotoxic T-cell function (18, 45, 46). However, the interaction between T cells and antigen-presenting cells (APCs) under CRT plus immunotherapy is little known. We here illustrated the spatial connection of these two types of immune cells when adding PD-1 blockade to CRT in ESCC patients. An accordant distribution pattern between T cells and DCs/macrophages in the 100-μm distance to tumor cells throughout the combination treatment as well as their association with patient survival in our findings was observed. Close spatial positions facilitated the signal transmission between these two types of cells. The T-cell and APC crosstalk was a double-edged sword in immune response, resulting in immune activation as well as anergy or tolerance (47, 48). These lines of evidence as well as our findings supported the vital role of the mutual regulation and influence among T and APC subsets in the tumor immune microenvironment both during tumor progression and during therapeutic interventions. Additionally, we found that the relevance between DCs and macrophages became even higher after the combination treatment. These two myeloid-derived subsets either acted as suppressors or activators in tumor immune responses (49, 50). High inflexible plasticity, one of the most intriguing features in myeloid-derived cells, led to diverse subgroups in these cells and their sensitive and rapid response to the internal and external signaling from the tumor microenvironment (51, 52). More precisely, identifying the subgroups of these myeloid-derived cells and their association with other immune cells, stromal cells, and tumor cells would provide important clues that will help interpret the mechanisms and treatment outcome of CRT plus immunotherapy.

The present study and our previous study (18) showed a significant association between higher TMB and more T cells, DCs, and macrophages that accumulated near the tumor cells at baseline. Tumors with high TMB probably had high tumor neoantigens that induced the antitumor immune response (53). On neoantigens presented by APCs, tumor-specific T cells underwent clonal expansion with significant correlation between high TMB, high TCR Simpson, and low Shannon index in ESCC patients (38). High levels of TMB not only facilitated the tumor infiltration and activation of T cells but also optimized the spatial distribution of these cells in the tumor microenvironment. However, we (3, 4) and others (54) failed to connect the TMB and survival in RT or even adding immunotherapy in ESCC. Other factors that regulated the tumor immune microenvironment under CRT combined with PD-1 blockade contributed to the treatment outcome aside from the TMB effect.

Lastly, we firstly reported that smoking history was associated with a shorter distance of PD-1^−^CD4^+^ T cells to tumor cells in ESCC. Smoking is a major risk factor resulting in ESCC. The serum metabolites of cigarette (55), lncRNA H19 (56), and p53 mutation or overexpression (57, 58) induced by smoking exposure were closely correlated with ESCC incidence. Mechanically, smoking facilitated ESCC by the Y-linked LINC00278/Yin Yang 1 (YY1)-binding micropeptide/YY1/androgen receptor signaling pathway (59). However, whether smoking exposure attracted T-cell accumulation in tumor tissues is still unknown. The activation of the NF-κB pathway and alteration in metabolites, tumor, and stromal cells induced by exposure to smoking might affect the tumor immune microenvironment (60).

Several limitations existed in our study. As expected in phase Ib clinical studies, the sample size of this study was also small. Control groups, such as samples from standard CRT or PD-1 blockade alone, were not available. Functional evaluation was not applied in the study because of the limitation of ESCC tissues collected under endoscopic ultrasonography. In addition, we did not identify the subgroups of tumor-infiltrating immune cells in the present exploration study. Our ongoing phase III study (NCT04426955) of CRT combined with camrelizumab is under follow-up, which would help provide more evidence to strengthen the findings of this study.

In conclusion, we firstly illustrated the precise spatial distribution of tumor-infiltrating T cells in ESCC patients who received CRT combined with PD-1 blockade. Combining CRT and PD-1 blockade could improve the inhibitory status of tumor-infiltrating T cells, which could benefit the combination outcome. The characteristic distribution patterns of T cells, DCs, and macrophages could be promising predictive candidates for this combination treatment. Further studies on precision tumor fields would provide new mechanisms in immunotherapy in solid tumors.

## Data availability statement

The original contributions presented in the study are included in the article/**Supplementary Material**, further inquiries can be directed to the corresponding authors.

## Ethics statement

The studies involving human participants were reviewed and approved by The institutional review board and ethics committee at Tianjin Medical University Cancer Institute and Hospital (E2018142). The patients/participants provided their written informed consent to participate in this study.

## Author contributions

WZ conceived and designed the study. CY, HH, ZZ, XM, and HW performed the exploration experiments. FC, TZ, XC, and JD collected the samples and clinical information. GZ, PT, HJ, MW, PW, and QP interpreted the data. WZ and CY drafted the manuscript. All authors contributed to the article and approved the submitted version.
