# Supplementary material for: Spatial distribution of tumor-infiltrating T cells indicated immune response status under chemoradiotherapy plus PD-1 blockade in esophageal cancer
Source: Front Immunol. 2023 May 19;14:1138054. doi: 10.3389/fimmu.2023.1138054 (PMC10235618; doi:10.3389/fimmu.2023.1138054)
Supplement: Supplementary file 1 [file DataSheet_1.docx]

**Supplementary file**

**Table S1. Samples available for exploration analysis**

| **Patient ID** | **FoundationOne CDx (Before the treatment)** | **Multiplex IF for Panel 1**  **(Baseline)** | **Multiplex IF for Panel 1**  **(on-treatment^a^)** | **Multiplex IF for Panel 2**  **(Baseline)** | **Multiplex IF for Panel 2**  **(on-treatment^a^)** | **Tumor mutation burden value** |
| --- | --- | --- | --- | --- | --- | --- |
| 1 | × | √ | × | √ | × |  |
| 2 | √ | √ | √ | √ | √ | 1 |
| 3 | √ | √ | √ | √ | √ | 4 |
| 4 | √ | √ | √ | √ | √ | 10 |
| 5 | √ | √ | √ | √ | √ | 6 |
| 6 | √ | √ | √ | √ | √ | 6 |
| 7 | √ | √ | √ | √ | √ | 6 |
| 8 | × | √ | × | √ | × |  |
| 9 | √ | √ | √ | √ | √ | 8 |
| 10 | × | ×^b^ | √ | ×^b^ | √ |  |
| 11 | √ | √ | √ | √ | √ | 8 |
| 12 | × | √ | √ | √ | √ |  |
| 13 | √ | √ | √ | √ | √ | 4 |
| 14 | √ | √ | √ | √ | √ | 29 |
| 15 | √ | √ | √ | √ | √ | 6 |
| 16 | √ | ×^b^ | √ | ×^b^ | √ |  |
| 17 | √ | √ | √ | √ | √ | 4 |
| 18 | √ | √ | √ | √ | √ | 4 |
| 19 | × | √ | √ | √ | √ | 6 |
| 20 | × | √ | √ | √ | √ |  |

^a^ after 40 Gy radiotherapy. ^b^stained tumor section without tumor cells included. IF=immunofluorescence.

**Table S2. Baseline patient characteristics**

| **Characteristic** | **Data (n=20)** |
| --- | --- |
| Age, median (range), y | 64 (56–74) |
| Sex |  |
| Male | 18 (90) |
| Female | 2 (10) |
| ECOG performance status score |  |
| 0 | 16 (80) |
| 1 | 4 (20) |
| Smoking status |  |
| Never | 7 (35) |
| Former or current | 13 (65) |
| Drinking status |  |
| Never | 6 (30) |
| Former or current | 14 (56) |
| Location |  |
| Cervical segment | 2 (10) |
| Upper thoracic segment | 7 (35) |
| Middle thoracic segment | 8 (40) |
| Inferior thoracic segment | 3 (15) |
| AJCC8^a^ disease stage |  |
| I | 1 (5) |
| III | 6 (30) |
| IV | 13 (65) |
| AJCC6^b^ disease stage |  |
| IIb | 2 (10) |
| III | 13 (65) |
| IVa | 5 (25) |

Data are n (%), unless otherwise specified. ^a^8^th^ (2017) version of the AJCC Staging Manual. ^b^6^th^ (2002) version of the AJCC Staging Manual. AJCC= American Joint Committee on Cancer. ECOG=Eastern Cooperative Oncology Group.


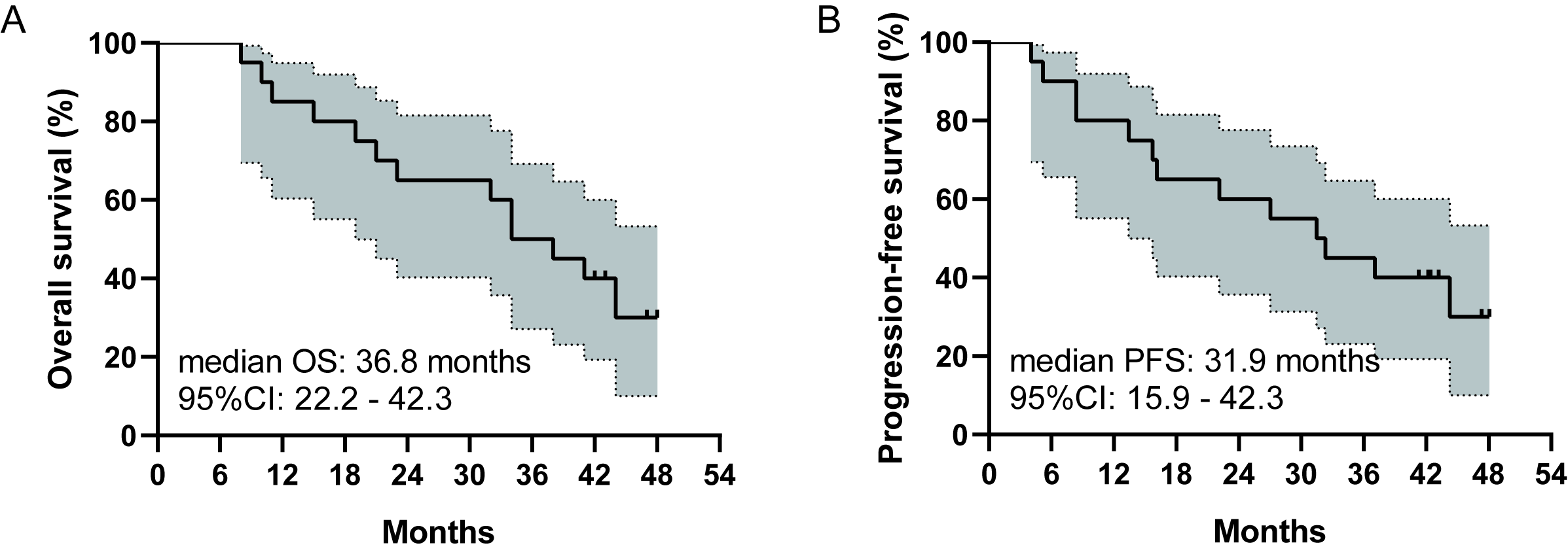


**Figure S1. Kaplan–Meier curves showing overall and progression-free survival of ESCC patients receiving chemoradiotherapy combined with anti-PD-1 antibody camrelizumab as first-line treatment.**

**Figure S2. Kaplan–Meier curve showing progression-free survival based on CD4^+^ T cells in the tumor compartment at baseline.**

Cutoff value, 13.97%.


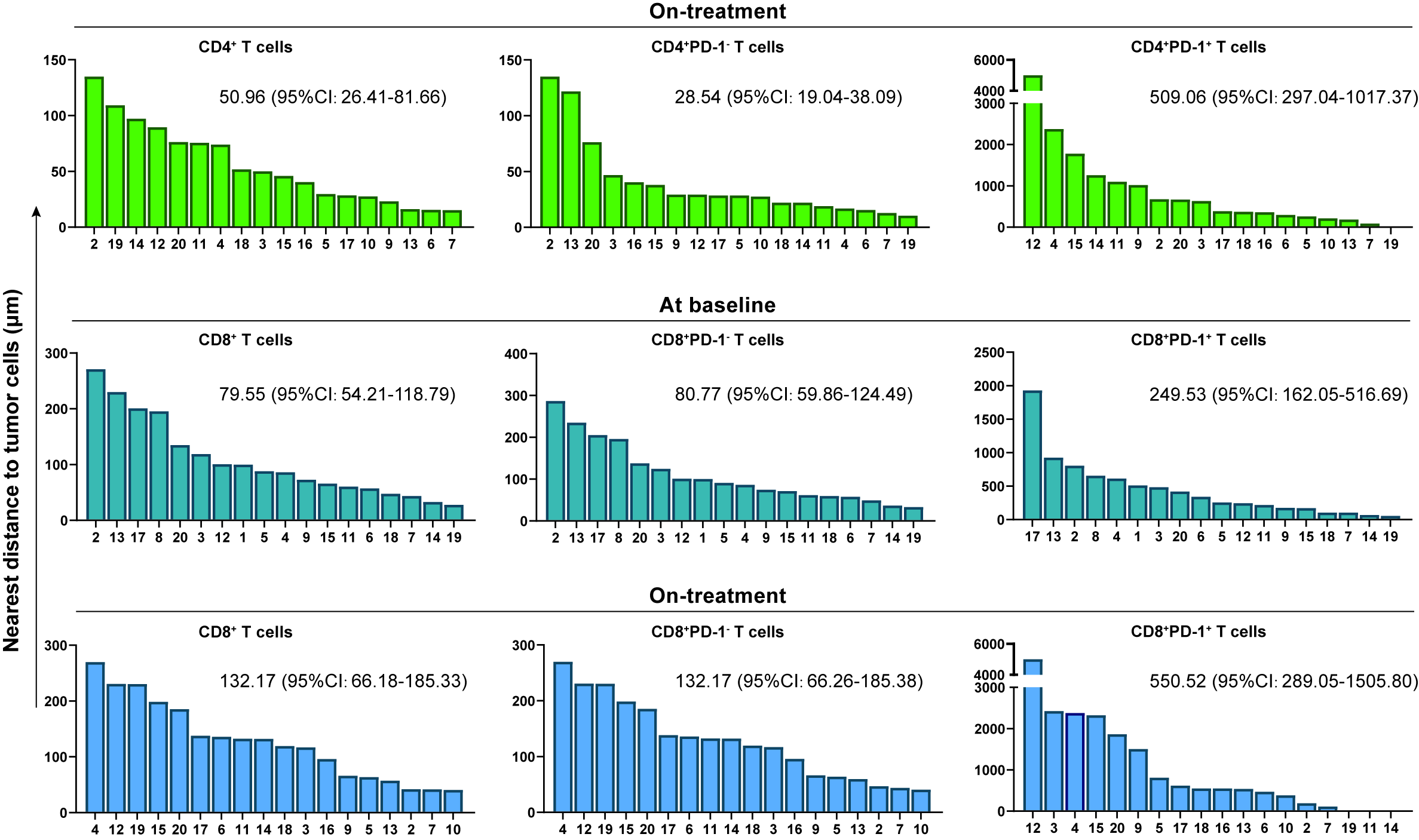


**Figure S3. Nearest distance of T-cell subsets to tumor cells.**

Statistics: median (95% confidence interval). CI, confidence interval. No., patient ID.


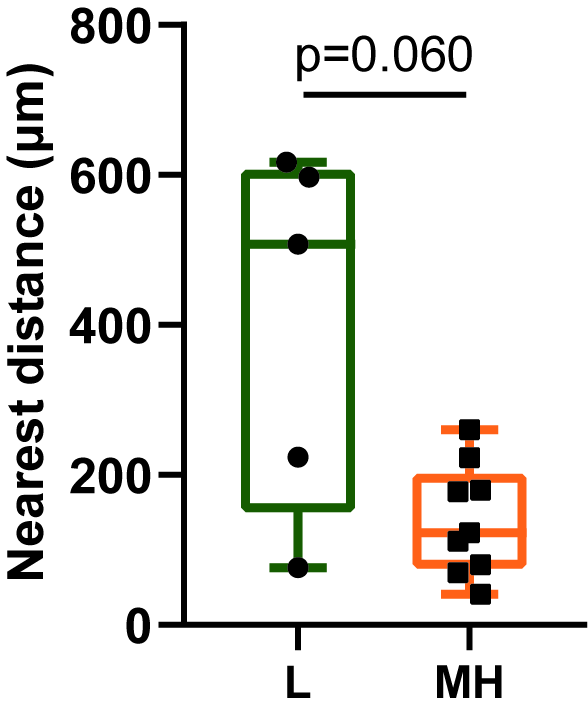


**Figure S4. Nearest distance of CD4^+^PD-1^+^ T cells to tumor cells between patients with low and middle/high TMB at baseline.**

L, TBM low (≤ 5 TMuts/Mb). MH, TBM middle (6-19 Muts/Mb) or high (**≥** 20 Muts/mb). TMB, tumor mutation burden.


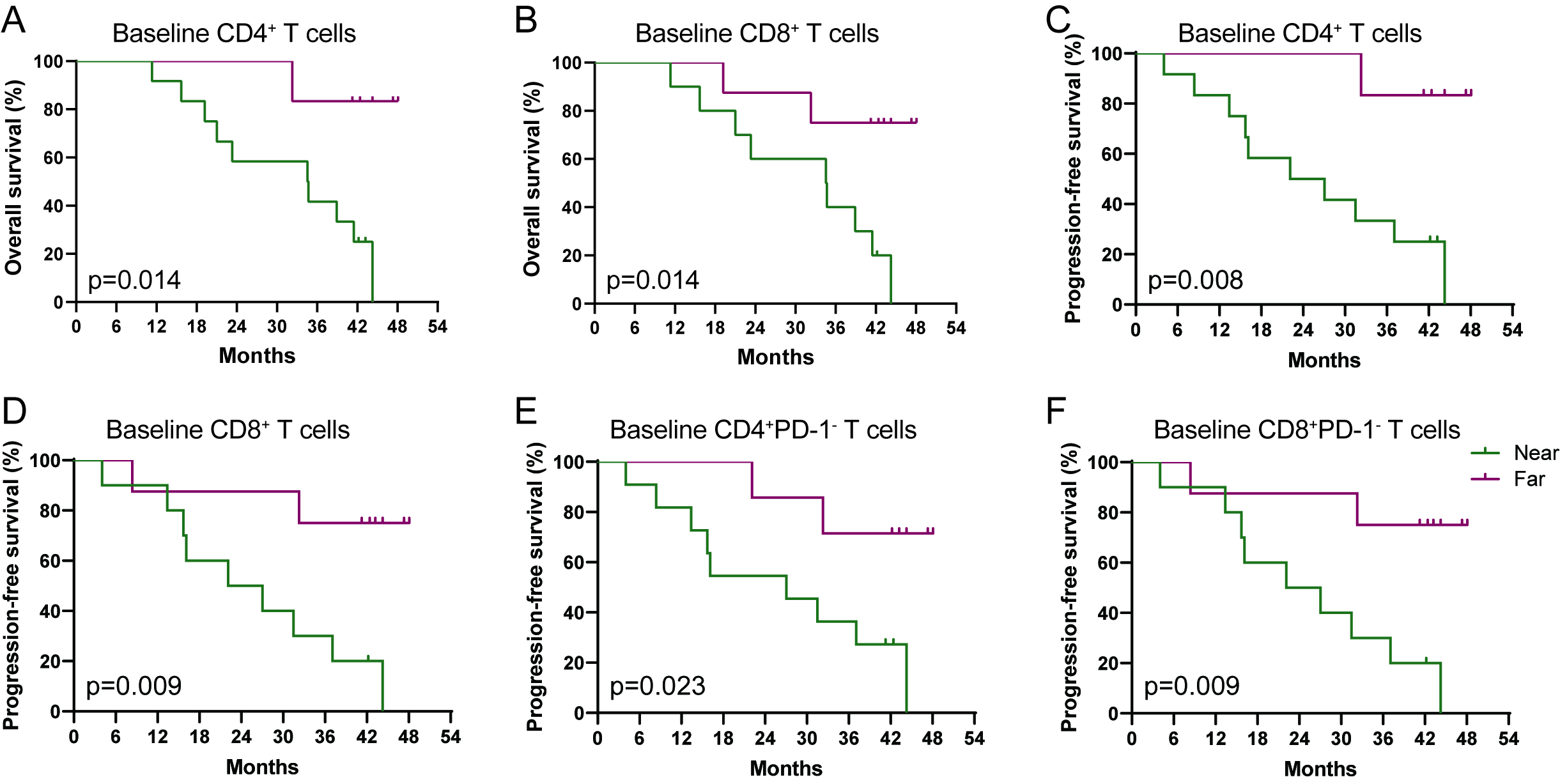


**Figure S5. Kaplan–Meier curves showing overall and progression-free survival based on the nearest distance of baseline CD4^+^ T cells and baseline CD8^+^ T cells to tumor cells.**

Cutoff value, A-F: 57.40, 93.99, 57.40, 93.99, 79.58, and 95.88 μm.


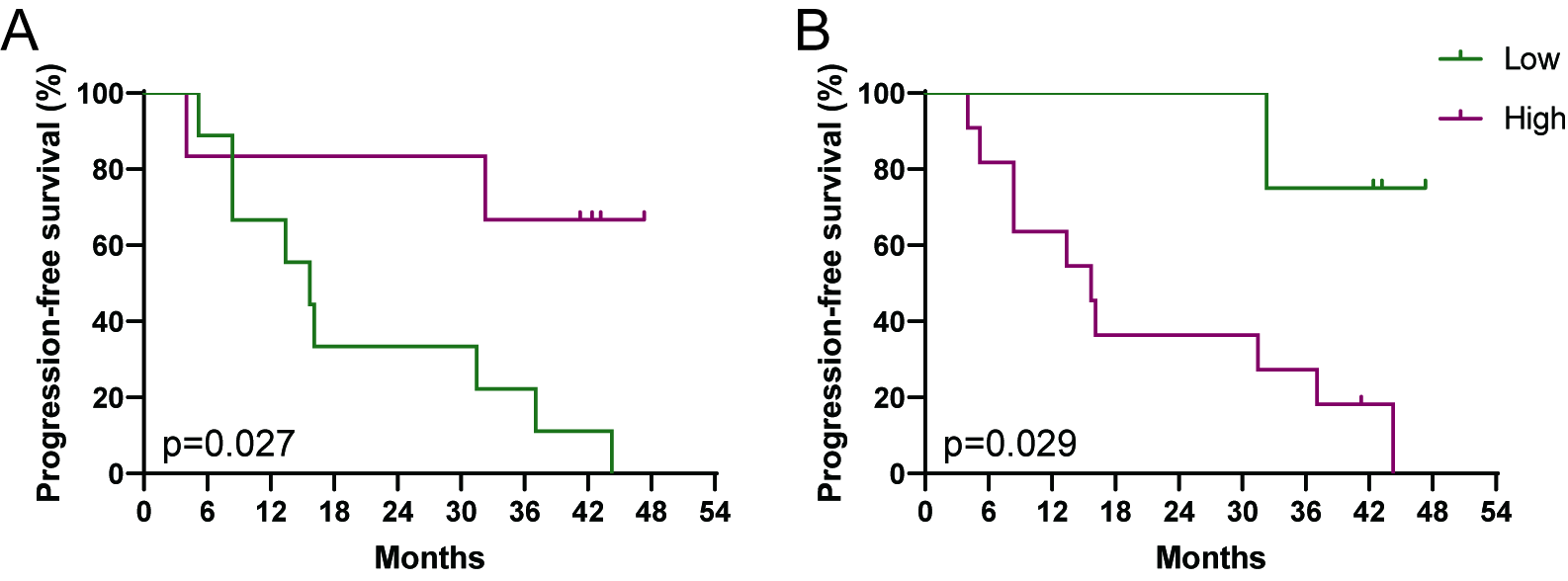


**Figure S6. Kaplan–Meier curve showing overall survival based on the proportion (A) and average distance (B) of CD8^+^PD-1^+^ T cells within 100 μm distance to tumor cells during treatment.**

Cutoff value, 38.72% for proportion of CD8^+^PD-1^+^ T cells; 47.75 μm for average distance of CD8^+^PD-1^+^ T cells.


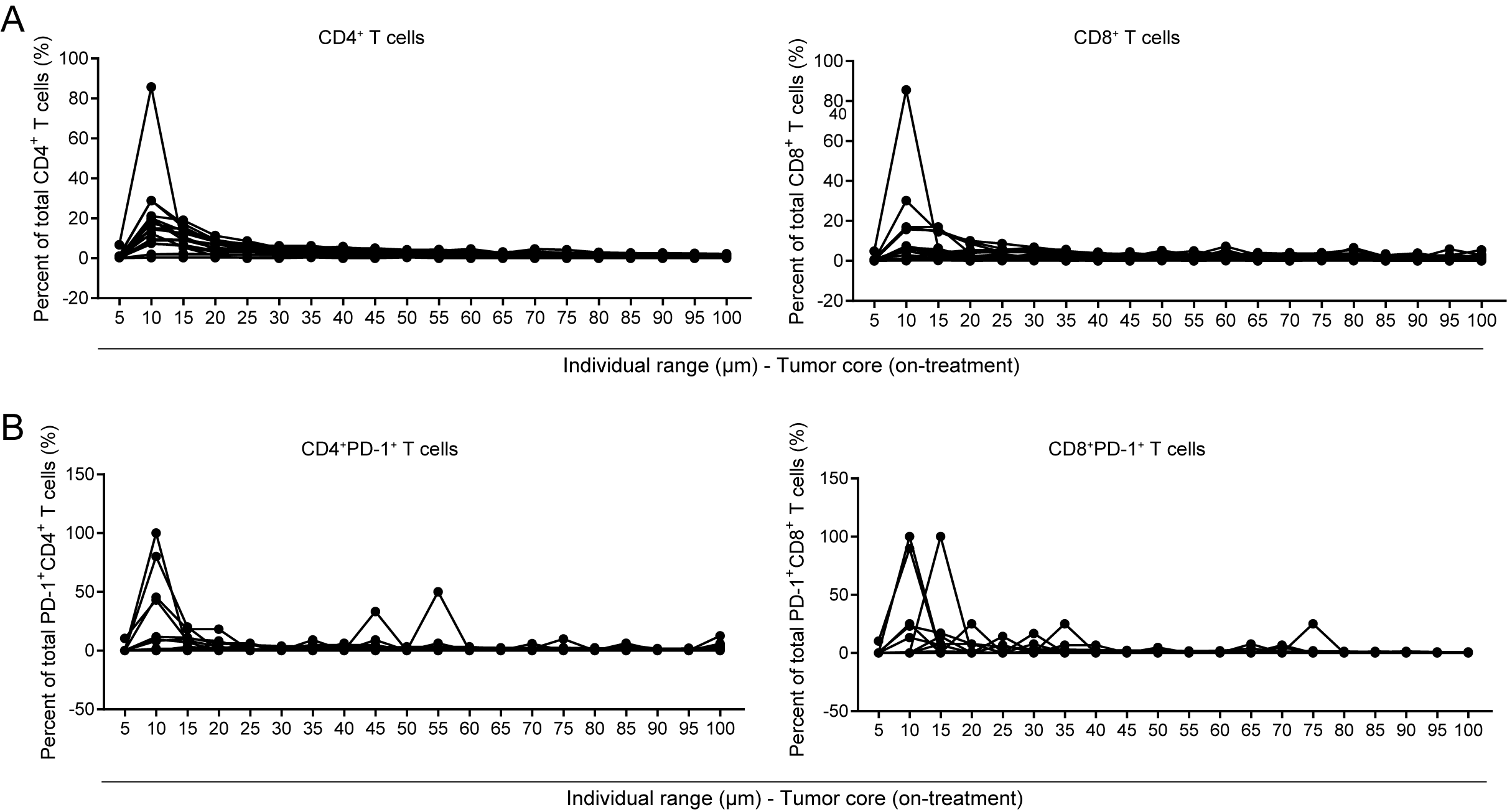


**Figure S7. Proportion of T-cell subsets in each 5 μm range across 100 μm distance to tumor cells after 40 Gy radiotherapy.**


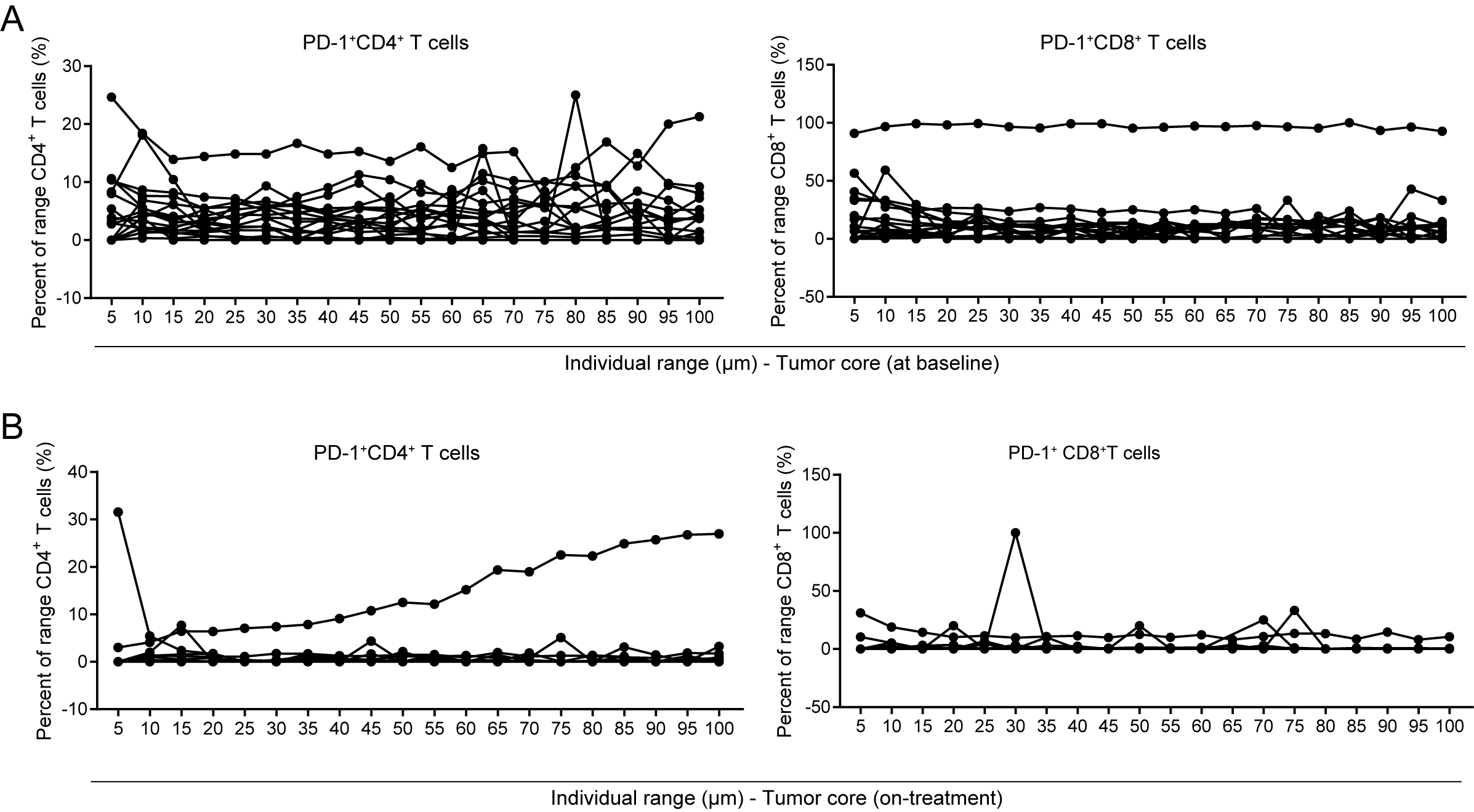


**Figure S8. Ratio of PD-1^+^ T cells in T cells in each 5 μm range across 100 μm distance to tumor cells at baseline and after 40 Gy radiotherapy.**


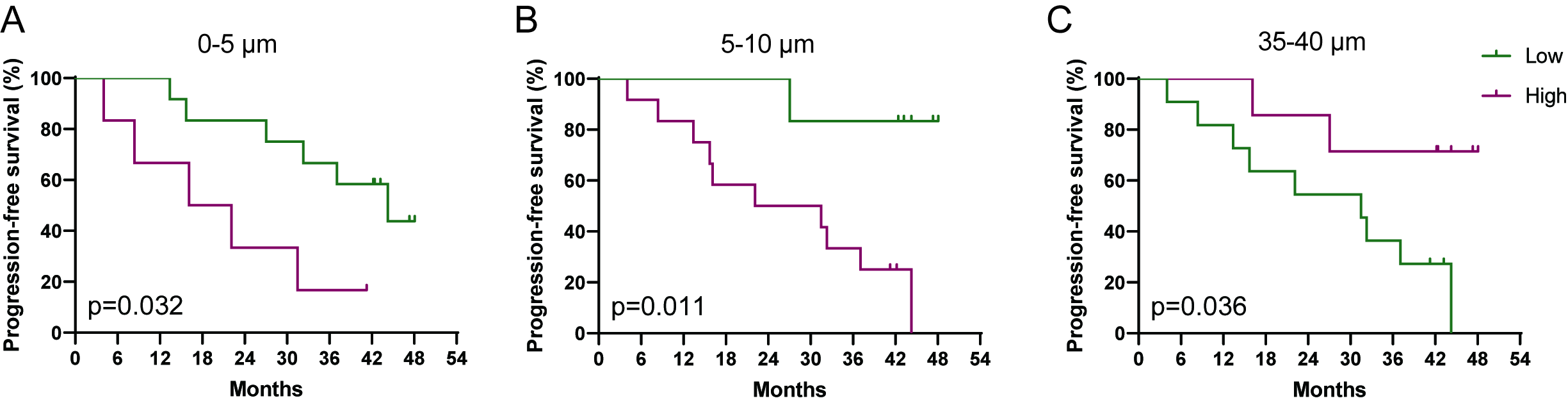


**Figure S9. Kaplan–Meier curve showing progression-free survival based on the proportion of CD4^+^ T cells in 0-5, 5-10, and 35-40 μm distance to tumor cells at baseline.**

Cutoff value, 0.97%; 14.95%; 3.81%.


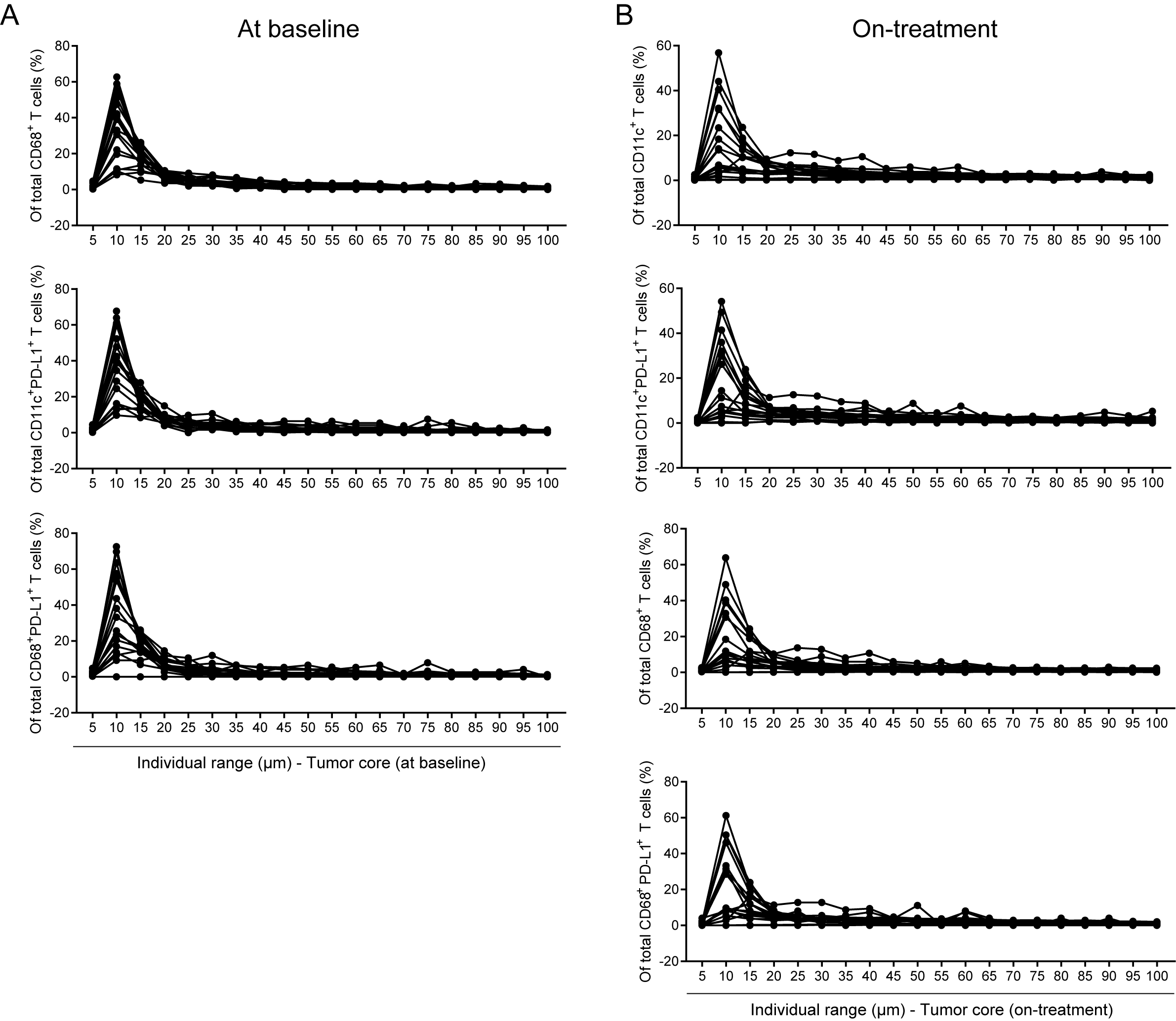


**Figure S10. Proportion of myeloid-derived cells in each 5 μm range across 100 μm distance to tumor cells after 40 Gy radiotherapy.**


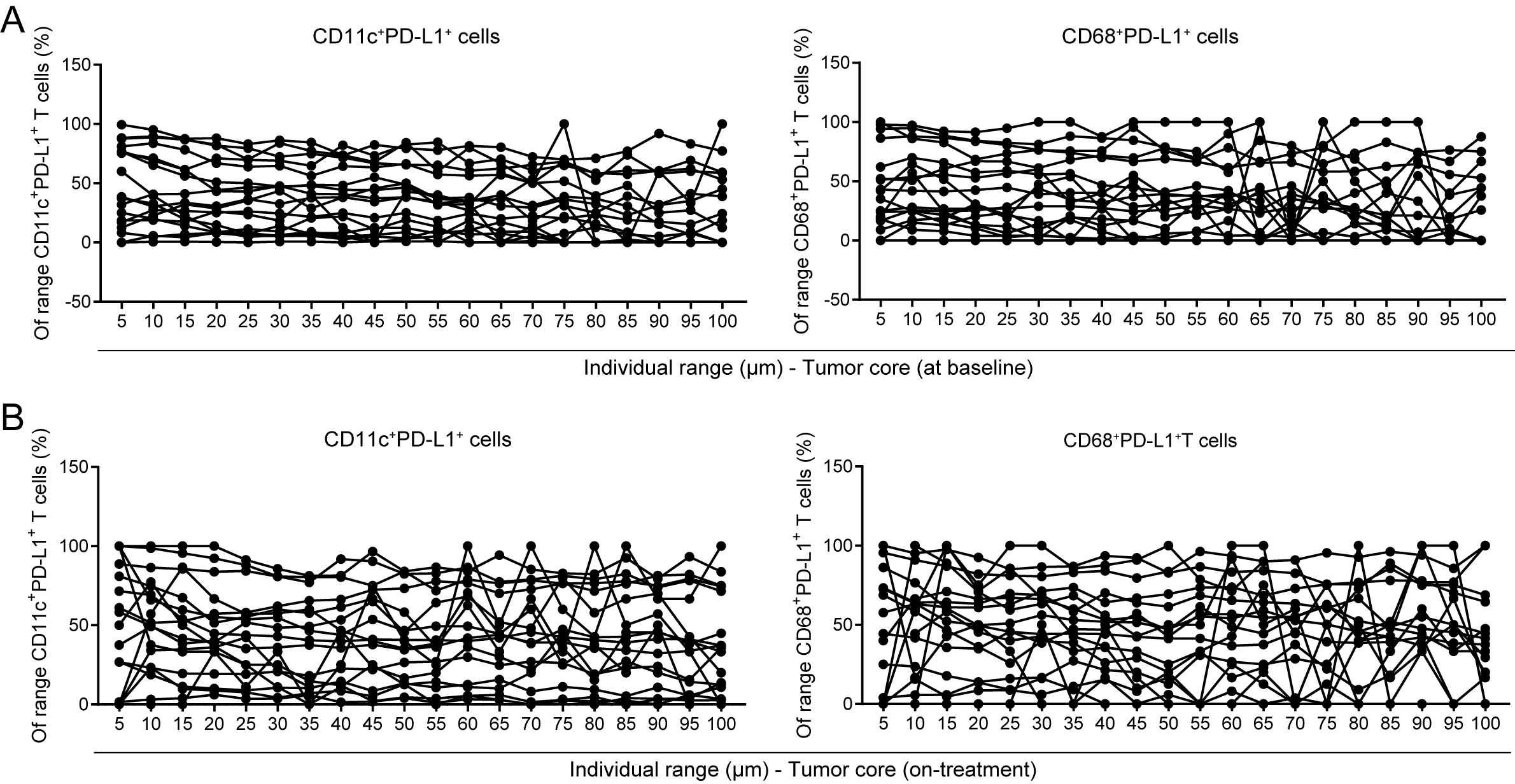


**Figure S11. Ratio of PD-L1^+^ dendritic cells and macrophages in dendritic cells and macrophages in each 5 μm range across 100 μm distance to tumor cells at baseline and after 40 Gy radiotherapy.**


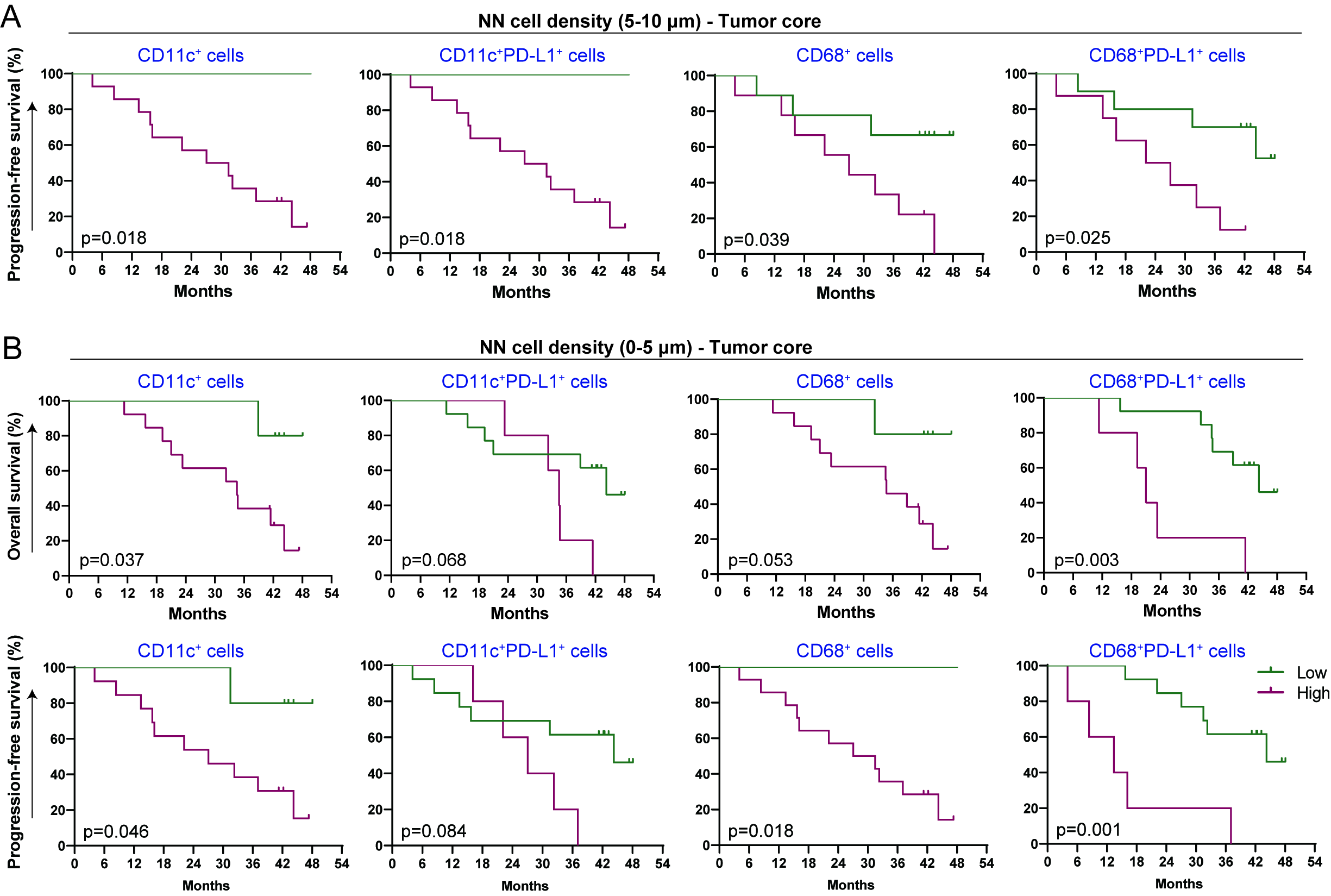


**Figure S12. Kaplan–Meier curve showing overall and progression-free survival based on the proportion of myeloid-derived cells in 0-5 and 5-10 μm range distance to tumor cells at baseline.**

Cutoff value, 18.79%, 16.14%, 20.91% and 40.89% in 5-10 μm distance; 0.83%, 2.36%, 0.63% and 2.04% in 0-5 μm distance.


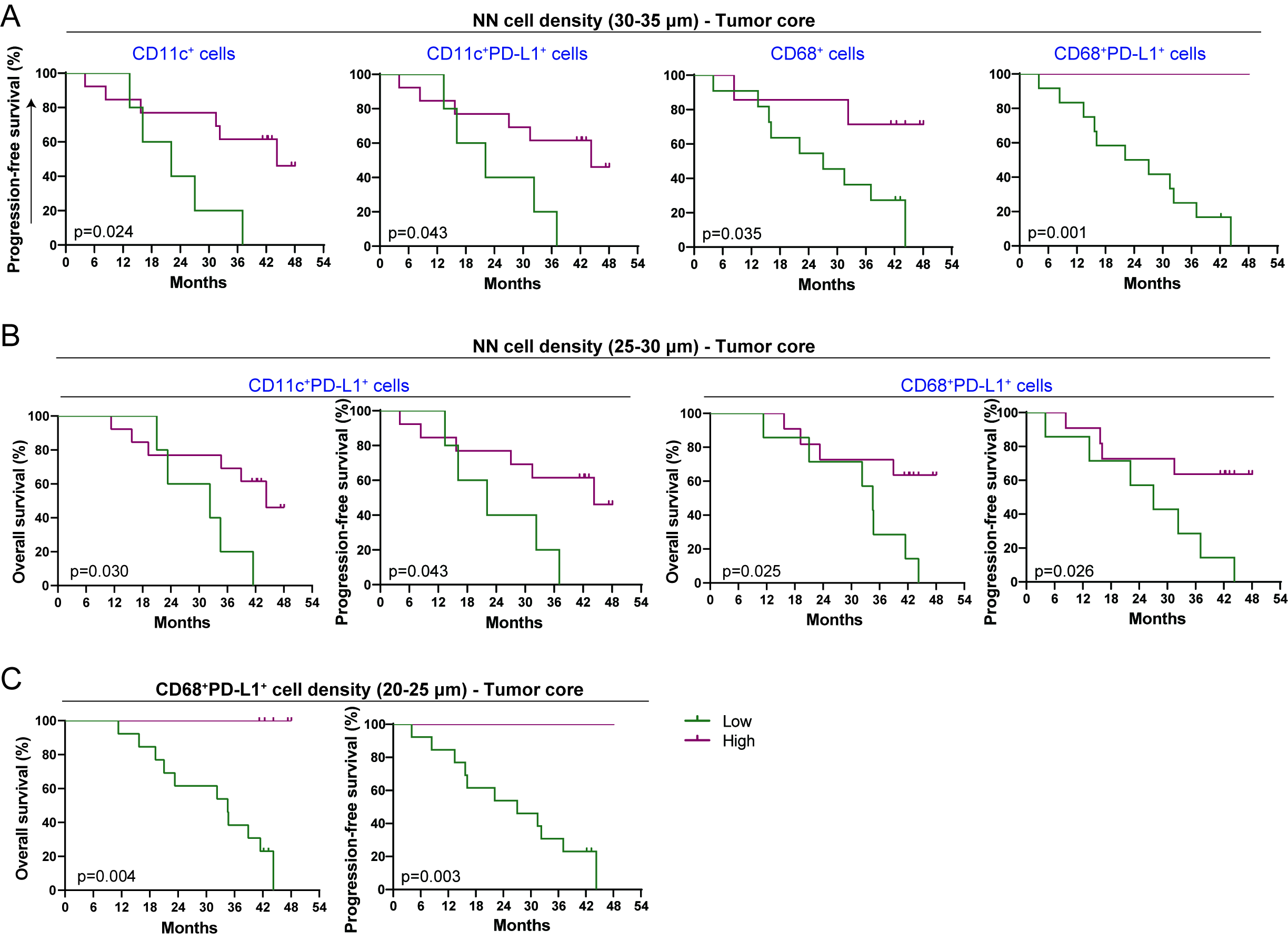


**Figure S13. Kaplan–Meier curve showing overall and progression-free survival based on the proportion of myeloid-derived cells in 20-25, 25-30 and 30-35 μm range distance to tumor cells at baseline.**

Cutoff value, 2.75%, 2.11%, 3.55% and 3.40% in 30-35 μm distance; 3.20% and 2.55% in 25-30 μm distance; 6.23% in 20-25 μm distance.
